# Training nurses in auriculotherapy/auricular acupuncture: possible paths for Portugal

**DOI:** 10.1590/1980-220X-REEUSP-2025-0397en

**Published:** 2026-01-26

**Authors:** Daiana Cristina Wickert, Diéssica Roggia Piexak, Daniela Dallegrave, Tereza Maria Mendes Diniz de Andrade Barroso, Maria Denise Schimith

**Affiliations:** 1 Universidade Federal de Santa Maria, Programa de Pós-Graduação em Enfermagem, Santa Maria, RS, Brazil.; 2 Universidade Federal do Rio Grande, Programa de Pós-Graduação em Enfermagem, Rio Grande, RS, Brazil.; 3 Universidade Federal do Rio Grande do Sul, Escola de Enfermagem e de Saúde Coletiva, Departamento de Assistência e Orientação Profissional, Porto Alegre, RS, Brazil.; 4 Universidade de Coimbra, Escola Superior de Enfermagem, Unidade de Investigação em Ciências da Saúde-Enfermagem, Coimbra, Portugal.

**Keywords:** Auriculotherapy, Acupuncture, Ear, Nursing, Teaching, Public Health

## Abstract

**Objective:**

To report on the experience of developing training in auriculotherapy/auricular acupuncture for nurses in Portugal.

**Method:**

Descriptive experience report, using the methodological framework proposed by Donabedian and informed and dialogued descriptive analysis. The training carried out is part of a thesis.

**Results:**

The experience took place from January to February 2025, during academic mobility at the Coimbra School of Nursing. This is a collective training course, developed with 19 nurses working in Portugal and with no previous training in auriculotherapy, who were intentionally invited. The course was adapted following the proposed guidelines for training nurses in auriculotherapy/auricular acupuncture, with educational objectives based on Bloom’s Taxonomy. The course consisted of 75 hours of online theory. The practical workload was tested, varying from five to 20 hours.

**Conclusion:**

The experience is unprecedented and innovative and may support the regulation of training in auriculotherapy/auricular acupuncture as an intervention by nurses, based on the Brazilian model.

## INTRODUCTION

Traditional, Complementary, and Integrative Medicines (TCIM), as designated by the World Health Organization (WHO), are practices, products, and knowledge used by different peoples and cultures around the world. Internationally, TCIMs receive different names, one of which is Complementary and Alternative Medicine (CAM)^(1)^.

In the Brazilian context, TCIM or CAM are called Integrative and Complementary Health Practices (PICS in the Portuguese acronym), which in 2006 were consolidated through the National Policy on Integrative and Complementary Practices (PNPIC) in the Unified Health System (SUS), an important milestone for the advancement of the 29 practices recognized in the SUS^(2)^, of which auricular acupuncture is included in the scope of acupuncture/Traditional Chinese Medicine (TCM) practices^(3)^. Due to the diversity of nomenclatures that vary according to the branch or school, this article will use the term auriculotherapy/auricular acupuncture to refer to auricular therapy, in an attempt to ensure greater coverage.

In Portugal, TCIMs are commonly known as non-pharmacological therapies or interventions (NPIs)^(4)^. The term Non-Conventional Therapies (NCTs) is also used, defined as “Therapies that are based on a philosophical basis different from conventional medicine and apply specific diagnostic processes and their own therapies.” In 2003, acupuncture, phytotherapy, homeopathy, naturopathy, osteopathy, and chiropractic were recognized in the country (Law No. 45/2003), and in 2013, TCM was recognized (Law No. 71/2013)^(5)^. Furthermore, the term Complementary and Alternative Therapies (CAT) is also used in the context of Portuguese nursing^(6)^.

Nursing, which focuses on care, can use auriculotherapy as a tool to promote autonomy by including the intervention in its care practice^(7)^. A recent methodological study constructed and validated the content of the components of nursing intervention in auriculotherapy, thus contributing to the possible inclusion of auriculotherapy as a nursing intervention in the Nursing Interventions Classification (NIC), which demonstrates an advance in standardized language in clinical practice^(8)^.

In terms of regulation, Resolution No. 739 of February 5, 2024, of the Brazilian Federal Nursing Council (COFEN) stands out, recognizing auriculotherapy as training through free courses, recommending a minimum workload of 80 hours^(9)^, but without further guidance. The resolution presents acupuncture separately from auriculotherapy, as a specialization of at least 360 hours. In Portugal, acupuncture is a degree^(10)^.

In Portugal, although there is no specific regulation by the Order of Nurses for the use of auriculotherapy, institutional documents have shown the use of practices with the same principle in the context of nursing. A Master’s Report in Maternal Health Nursing and Obstetrics from the Lisbon School of Nursing, which uses acupressure as a nursing intervention, points out that the Board of the College of Maternal and Obstetric Health Specialists recommends the use of non-pharmacological methods for pain relief during labor, and that acupressure is among the recommended techniques. The author describes that acupressure is based on the same principles as acupuncture, but that needles are replaced by the application of pressure, which is beneficial in that it makes it a non-invasive technique without the need for specific equipment^(6)^.

Thus, the authors reinforce the independent nature of auriculotherapy/auricular acupuncture training offered as a free course or professional qualification. The authors understand that, because it studies only the ear microsystem, auriculotherapy is not a practice dependent on acupuncture^(11)^, that is, training in systemic acupuncture is not necessary to learn auriculotherapy/auricular acupuncture.

Nursing work is often associated with the logic of PICS. However, today, there are no guidelines with minimum parameters for the training of nurses in auriculotherapy/auricular acupuncture.

As a result of a national survey on the training and performance of nurses in PICS in Brazil, through a doctoral thesis linked to the study, a training guideline for nurses in auriculotherapy/auricular acupuncture (ISBN No. 978-65-01-86051-0) was developed, which was used as the basis for adapting the training course that supports this experience report.

In this sense, the justifications for this experience report are presented:

The qualification of nurses’ training reflects health care for a large contingent of people, given that the most significant number of health workers is in nursing. Nursing is present throughout the world, in the most diverse places.

The study proposes the gathering of elements to support the professional training of nurses in auriculotherapy/auricular acupuncture. At the same time, this systematization can cover or be applied to other very similar or related areas, such as other TCM or other health professions.

Auriculotherapy/auricular acupuncture is performed by nurses in a significant way. Patient safety, the adoption of evidence-based practices, and effective nursing care are expected results with improved professional training, one of the objectives of the Global Strategy for TCM 2025–2034^(12)^.

Thus, the objective is to report on the experience of developing training in auriculotherapy for nurses in Coimbra, Portugal.

## METHOD

### Study Design

This is a descriptive report of an experience presenting training in auriculotherapy/auricular acupuncture developed with Portuguese nurses. This type of study describes an innovative experience in training that is widely disseminated in the context of Brazilian nursing but still scarce in Portugal.

The focus of the experience is research, given that the training is part of a thesis project that aims to develop guidelines for training in auriculotherapy/auricular acupuncture based on WHO benchmarks. It is hoped that the experience will foster new projects that address training.

It should be noted that there are no guidelines with recommendations for writing experience reports; however, we chose to follow the assumptions for the preparation of experience reports as scientific knowledge proposed by Mussi et al.^(13)^.

### Setting

The experience took place in Coimbra, Portugal, during advanced training (academic mobility) at the Coimbra School of Nursing (ESEnfC). Coimbra is a Portuguese city located in the central region of the country.

The venue was the Order of Nurses (OE) of Coimbra, in a room provided for the training. The OE, created in 1998, is the public professional association that incorporates all nursing professionals working in Portugal, deliberating on the regulation of nurses and specialist nurses. The OE in Coimbra is located on the same street as *Polo A* of the ESEnfC. It is an accessible building, which provided the structural and technological support necessary for the smooth running of the training.

ESEnfC was created in 2006 from the merger of the Dr. Ângelo da Fonseca Higher School of Nursing (founded in 1881) and the Bissaya Barreto Higher School of Nursing (founded in 1971). ESenfC has three campuses and a Health Sciences Research Unit: Nursing (UICISA: E), which offers a bachelor’s degree in Nursing, 11 master’s degrees, six postgraduate courses, and a doctoral program, with a total of more than 1,900 students enrolled at the institution. It is currently undergoing a process of integration with the University of Coimbra and will be formally integrated by January 2026, according to Decree-Law No. 83/2024 (https://files.diariodarepublica.pt/1s/2024/10/21200/0001700023.pdf).

### Process Description

The results described in this study are the result of planning and conducting a training course in auriculotherapy with nurses in Portugal. Specifically, the course was conducted from January to February 2025.

### Data Analysis

To describe the experience, we used the methodological framework proposed by Donabedian^(14)^, divided into:

(1) Structure: we sought to evaluate the available resources, such as the qualifications of the teachers, the infrastructure of the OE, and the teaching materials used.
(2) Process: conducting the training, teaching methods, and practical activities.
(3) Results: evaluating the effects of the training, the ability to apply auriculotherapy, and the impacts on society.

The choice of the reference framework was made because it allowed for a systemic and organized view of all aspects surrounding the experience described. Even though this is not a health assessment, Donabedian’s triad proved to be adequate for describing the training of nurses in auriculotherapy/auricular acupuncture.

As a form of analysis, an informative and dialogical description will be carried out according to Mussi et al.^(13)^, considering that this is an unprecedented training linked to a research stage.

### Ethical Aspects

This experience report was derived from activities carried out during an academic mobility program that includes a stage of doctoral research in nursing, approved by the Ethics Committee of the Portuguese Society of Mental Health Nursing (CE-ASPESM) and by the Research Ethics Committee of the Federal University of Rio Grande do Sul (UFRGS), under Certificate of Presentation for Ethical Appraisal (CAAE) No. 43306921.6.0000.5347.

## RESULTS

### Structure for Conducting the Training

The partnership between ESEnfC faculty and Brazilian researchers stems from previous projects, as well as a mutual interest in bringing training in auriculotherapy/auricular acupuncture to nurses in Portugal, based on Brazilian experience in this area.

Thus, planning to make the training possible began with the submission of a doctoral project to compete for the Rio Grande do Sul State Research Support Foundation (FAPERGS) call for proposals number 04/2024 – Emergency aid for the mobility of doctoral students due to the climate emergency in Rio Grande do Sul (RS), launched on June 21, 2024, for which the authors applied and were accepted. The purpose of the call for proposals was to provide financial support for the mobility of doctoral students regularly enrolled in postgraduate programs at institutions based in the state of RS, Brazil. Academic mobility, in the case of this report, comprised a period of activities directly related to the project at ESEnfC in Portugal.

The scientific and methodological basis for the training proposal developed in Portugal, in addition to clinical practice, comes from the analyses and discussions arising from the EnfPICS Research (National survey on the educational and professional profile of integrative health nurses and traditional practices), developed by Brazilian researchers who have been deepening their knowledge about the profile and training of nurses in auriculotherapy. In total, nine articles and a book chapter were published. Lectures have also been given at events in the field, such as the IV CONGREPICS, the IV Conference on Integrative and Complementary Health Practices at the University of São Paulo (USP), 3^rd^ World Congress on Traditional, Complementary and Integrative Medicine, the 16th International Congress of the United Network, at which the 1st National Workshop for the development of training guidelines for nurses in PICS: auriculotherapy was held, giving rise to the draft proposal for guidelines for the training of nurses in auriculotherapy/auricular acupuncture, which guided the training that will be reported on.

The teaching materials used were adapted from the extension course promoted by the #SUStentaPICS Extension Program (School of Nursing and Collective Health – UFRGS), in partnership with the State Health Secretariat of Rio Grande do Sul (PICS Technical Area) and with the support of ABENAH, entitled “Professional qualification in PICS: auriculotherapy.” The course was adapted following the proposed guidelines for training nurses in auriculotherapy/auricular acupuncture.

The adaptation of the course was approved by the Extension Commission (COMEX) of the School of Nursing and Public Health at UFRGS and the Extension Chamber (CAMEX) at UFRGS. The course had an online theoretical stage, via UFRGS Moodle, and a practical stage in person at OE in Coimbra, Portugal. The materials for the practical class were donated and delivered to the 19 nurses participating in the course.

The course was taught by two professionals with training in free courses in auriculotherapy. One of them has been a primary care nurse in Brazil for three years, has a doctorate in nursing, and is a member of the Brazilian Association of Acupuncture Nurses and Nurses in Integrative Practices (ABENAH) and the Brazilian Academic Consortium of Integrative Health (CABSIN). and the other, a doctor of nursing, a professor at the Federal University of Rio Grande (FURG) for ten years, a member of CABSIN and the Working Group on Medical Rationalities and Integrative and Complementary Practices of the Brazilian Association of Collective Health. The professionals have daily clinical experience in the use of auriculotherapy/auricular acupuncture in nursing practice and have been developing unprecedented research in the area as researchers at EnfPICS.

### Training Development Process

The processes carried out were outlined considering the stages described in the training plan based on eight learning objectives defined in light of Bloom’s Taxonomy:

OBJECTIVE 1 – Develop and encourage actions favorable to Permanent Health Education (PHE), with the aim of ensuring the training and updating of the nursing team in the field of auriculotherapy/auricular acupuncture and its derivatives;

OBJECTIVE 2 – Use the concepts and vision of the whole human being and welcoming care as a model for providing auriculotherapy/auricular acupuncture and its derivatives as a nursing intervention;

OBJECTIVE 3 – Provide consultation and nursing care based on the principles governing the practice of auriculotherapy/auricular acupuncture and its derivatives;

OBJECTIVE 4 – Indicate, prescribe, and implement auriculotherapy/auricular acupuncture and its derivatives in the provision of nursing care in any health service, in the private and public spheres, recognizing it as a strategy that contributes to the promotion of the individual’s overall health, associated with other care and medical rationalities;

OBJECTIVE 5 – Perform nursing diagnoses based on taxonomies recognized by the category, establish related factors and defining characteristics, and include auriculotherapy/auricular acupuncture and its derivatives as a nursing intervention (auricular acupuncture and auriculotherapy are not nursing interventions recognized by taxonomies, which is a gap to be filled. Currently, related interventions are: Skin stimulation and Acupressure);

OBJECTIVE 6 – Apply auriculotherapy/auricular acupuncture and its derivatives safely, considering health protocols, with different stimuli from invasive devices (needles and derivatives) and non-invasive devices (spheres, seeds, laser, acupressure, and derivatives);

OBJECTIVE 7 – Promote the development of scientific nursing knowledge, acting and coordinating teaching/lecturing, research, and training activities in auriculotherapy/auricular acupuncture and its derivatives;

OBJECTIVE 8 – Coordinate, plan, organize, and guide the nursing team in the implementation of auriculotherapy/auricular acupuncture and its derivatives in nursing care;

Therefore, considering the learning objectives, the course was divided into six learning centers (five theoretical and one practical):

Learning Core 1 (15 hours) – Auriculotherapy: presentation of training in auricular therapy. Presentation of the course; Experiences of integrative and complementary practices in the USA, Latin America, Eastern countries, and Europe; Introduction to Auriculotherapy.

Learning core 2 (15 hours) – Auriculotherapy: Theoretical bases according to reflexology: Anatomical structures and auricular points; Assessment methods in auriculotherapy; Treatment methods.

Learning core 3 (15 hours) – Auriculotherapy: Theoretical bases of traditional Chinese medicine (TCM): Medical rationales; Auriculotherapy according to the fundamentals of TCM.

Learning core 4 (15 hours) – Auriculotherapy: Theoretical bases according to biomedicine: Neurophysiology; biomedical evidence and potential adverse effects; red lines.

Learning core 5 (15 hours) – Auriculotherapy in primary health care: Primary Health Care and Auriculotherapy; use of Auriculotherapy in the routine of primary health care; continuing education and Auriculotherapy in primary health care, as well as in pain consultations in the field of oncology.

Learning core 6 (5 to 20 hours) – Practical classes: Inspection and palpation of the auricle; fixing the seed to the auricle; clinical follow-up.

This is a collective training course developed with nurses working in Portugal who have no previous training in auriculotherapy/auricular acupuncture, these being the inclusion criteria. The class was composed of 19 Portuguese nurses. They were intentionally invited by a professor at ESEnfC to participate in the training. The participants were nurses already known to the professor for having completed specialization, master’s, or doctoral degrees at ESEnfC, or for working in services with which she has ties due to university classes and projects. A list of interested parties was created, containing the names, email addresses, and phone numbers of potential participants.

Initially, on January 3, 2025, an instant messaging group was created on mobile phones with the participants in order to facilitate communication and answer questions regarding registration on Moodle and the Extension Portal of the Dean of Extension (PROREXT) at UFRGS to begin the theoretical classes. In addition to the instant messaging group, communications also took place via email.

In the theoretical stage, the materials used were made available via UFRGS Moodle. The modules were released according to each student’s progress, as they completed the readings and answered the questions for learning assessment. The materials consisted of handouts, scientific articles, a link to the Virtual Health Library MTCI evidence map, evidence-based clinical recommendations on auriculotherapy, and videos presenting and demonstrating the materials needed for the practical class. Dialogue was constant, and questions were answered collectively and individually, according to the nurses’ needs, via message or email.

The OE auditorium was used for the practical stage. The tables and chairs were arranged in a circle to encourage constant dialogue among the nurses. Four days of practical classes were organized, according to the academic mobility period, namely: January 30, February 13, 20, and 27, from 1:00 p.m. to 6:00 p.m., totaling 20 hours of practice. Due to the release of health services, not all nurses were present on all days of practical classes, requiring a minimum of 5 hours for certification.

On January 30, the first practical class took place, which consisted of personal and professional introductions, answering questions about the theoretical modules covered so far (they had completed up to module 3), and familiarization with the materials and assembly of seed plates. At that time, each nurse indicated the dates they could attend the practical classes, and the schedule of activities was organized according to the group’s availability, as shown in Table 1.

**Table 1:** Schedule of supervised practical classes and number of participants – Coimbra, Portugal, 2025.

Date of the practical lesson	Number of participating nurses
January 30th	18 nurses
February 13th	13 nurses
February 20th	14 nurses
February 27th	5 nurses

For nurses who completed 15 hours of practical classes, the training focused on biomedicine, reflexology, and TCM. For the group that completed 10 hours of practical training, the approach focused on biomedicine and reflexology. Based on the Brazilian experience, the authors understand that it is possible to apply auriculotherapy based solely on biomedicine and reflexology with quality and safety with 5 hours of supervised practice due to the training basis of the nursing degree. In the case of TCM, 5 hours are considered minimal and introductory, requiring further study with professional practice. According to the experience with nurses in Portugal, 10 hours was assessed as the minimum necessary.

**Figure 1:**
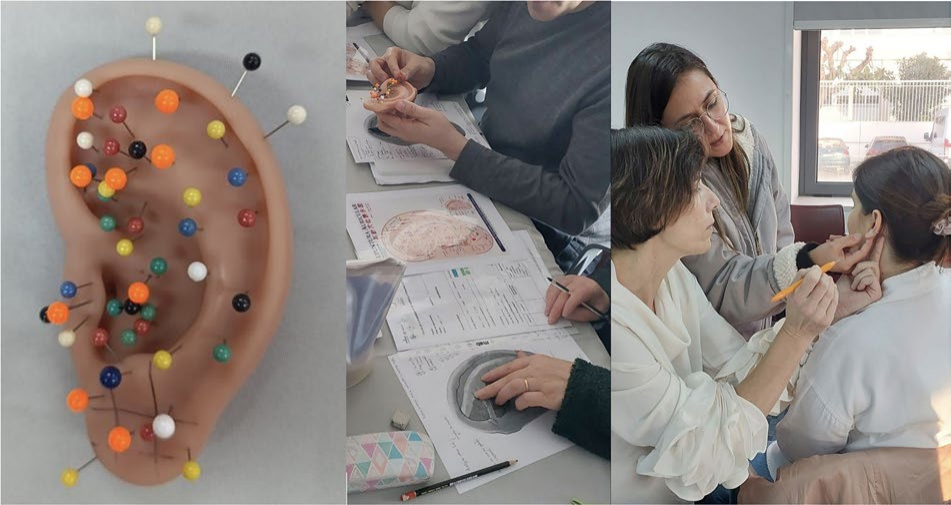
Identification of auricular points on a silicone model, drawing of the ear, and person – Coimbra, Portugal, 2025.

As a teaching strategy for locating auricular points, a silicone ear model and pins were used, with *in loco* location on the classmate’s ear and through the use of an ear drawing and auricular map, as shown in Figure 1.

Thus, the first part of the practice consisted of correctly locating the main auricular points. Afterwards, focusing on auriculotherapy and derivatives according to reflexology and biomedicine, the innervation of the ear (auricular temporal, vagus, and auricular major nerves) and the mechanism of action were presented. In order to learn the auricular inspection technique, the class was divided into pairs or trios, identifying changes in coloration, morphology, and vascularization in each other using spotlights. Then, the pairs practiced auricular palpation and point identification.

Afterwards, a clinical case was presented to simulate care, in which they had to locate the points according to biomedicine and reflexology and apply the points using seeds on their classmates.

In order too deepen their understanding of auriculotherapy/auricular acupuncture according to TCM, a book^(15)^ was used as a guide, addressing the different materials and their stimuli: harmonization (seeds, crystals, and silicon pads), tonification (1.0 to 1.5 mm needles, superficial needles, and gold balls), and sedation (1.8 to 2.0 mm needles, deep needles, and silver or stainless steel balls). Next, the theory of the five elements (seen in the theoretical stage) was revisited and two forms of treatment according to TCM were worked on: (1) Treatment of the shock element/organ and (2) Law of Generation and Dominance Cycles. Finally, simulations were carried out with clinical cases and treatment protocols in order to practice the three aspects studied.

### Training Results

After the training, in the form of a roundtable discussion, the training offered was evaluated, as well as how capable the nurses felt they were to apply auriculotherapy/auricular acupuncture. The feedback was positive regarding the quality of the classes and learning. As negative aspects, the need for more training hours for the use of auricular therapy according to TCM was mentioned. In addition, the form of regulation adopted by COFEN in Brazil and the paths towards possible regulation of professional practice by OE in Portugal were debated.The instant messaging group was maintained and discussions of cases, questions, and feedback frequently occur. As an impact of the training, it is expected that auriculotherapy/auricular acupuncture will spread in Portugal, thus fostering new classes and new horizons with regard to it as an intervention in nursing care.

## DISCUSSION

The term auricular acupuncture is part of the scope of TCM practices. There is no consensus on the origin of auricular therapy, which has been used for thousands of years in various countries, however, the greatest development of the technique and the first recorded documents are from China^(3)^. Auriculotherapy, according to Raphael Nogier’s recent manuscript, is a term derived from the studies of his father, Paul Nogier, a French physician^(16)^. The term auriculotherapy, in Brazil, is commonly used to refer to any aspect of the practice. It is worth noting that Paul Nogier’s studies boosted the recognition of auriculotherapy in the international context^(16)^. In view of the discussion in the scientific community about terminology, the choice to use the term auriculotherapy/auricular acupuncture aimed to encompass the different schools and branches of the practice, all with their scientific importance and relevance.

TCM is a medical rationality composed of a “complex web of interactions, with some moments of separation and others of approximation, between various schools and traditions that have emerged throughout history”^(17:104)^, and which is now practiced worldwide. Currently, TCM is subdivided into specialties (similar to the trend in biomedicine), a fact that may be influenced by Cartesian science. However, it should be remembered that in its origins, TCM therapy combined treatment modalities such as acupuncture, massage, pharmacopoeia, diet therapy, and therapeutic exercises^(17)^.

The most important document in the global context on acupuncture training is the WHO Benchmarks for the training of acupuncture, developed by the WHO in partnership with more than 50 experts from around the world. It establishes categories of acupuncture training, levels of content difficulty, and a curriculum for learning modules, which intersects TCM content with basic health disciplines^(18)^.

Nevertheless, the highlight in this discussion is the recognition of the multidisciplinary nature of acupuncture, in accordance with the WHO^(19)^, given that the document differentiates between the basic curriculum for biomedical professions, people who practice traditional medicine, and people without biomedical training^(18)^. Considering auricular acupuncture as a practice that is included in the scope of acupuncture, but that training can be carried out independently, through free courses (regardless of training in acupuncture), also reinforces its multidisciplinary nature. Evidence indicates that nurses working in the field of TCM have led to improvements in patients’ health status, among the interventions used is auriculotherapy/auricular acupuncture^(20)^.

A study that evaluated the training profile of auriculotherapy professionals in Brazil identified a multidisciplinary group of practitioners, most of whom were middle-aged women who worked in their private practices and had more than 40 hours of online training^(21)^. This aspect differs from Brazilian research that evaluated only nurses trained in auriculotherapy, most of whom concentrate their care in primary health care^(11)^.

In this sense, considering the Portuguese context, the European Social Survey, Round 7 (edition 2.0, 2014) identified that 14.1% of the population of Portugal uses some form of CAM for care. Almost a decade later, the European Social Survey, Round 11 (edition 2.0, 2023), showed that the typical profiles of CAM users in Europe remained stable: women, middle-aged groups, and people with high levels of education tend to use it more than other groups^(22)^.

The integrated practice of CAM alongside conventional medicine within the National Health Service is suggested in the 2019 Basic Health Law, in base 26, which states that “The practice of non-conventional therapies is regulated by law, carried out in an integrated manner with conventional therapies and in a way that ensures the protection of the health of individuals and communities, the quality of care, and based on the best scientific evidence”^(23:63)^.

A recent systematic review found that perceived usefulness, defined as the perceived benefits of an TCIM modality in meeting specific health needs or objectives, is the most influential factor in the use of TCIM, reported in 79% of studies. Thus, the authors suggest that by integrating widely accepted TCM practices, health authorities can ensure safe, standardized, and evidence-based use in medical settings. This integration can maximize patient engagement and satisfaction, leading to better health outcomes^(24)^.

Therefore, analyzing international experiences such as those in Brazil^(25)^, China, and the United States of America (USA) can foster implementation in other contexts. In China, TCM is the main practice, and efforts are focused on translating evidence and developing clinical practice guidelines, given that TCM has already been adopted at all levels of health care. In the USA, the healthcare system is dominated by conventional medicine, so implementation interventions focus on facilitating the provision of specific evidence-based TCIM modalities through referrals from conventional clinicians^(26)^.

In Brazil, given the potential and weaknesses in the implementation of the PNPIC, it is necessary to enable the training and qualification of an adequate number of professionals to work in the SUS. In this sense, authors affirm that the interest of professionals in training and the considerable number of teaching and research initiatives in public universities can catalyze this process^(25)^.

Currently, research is also focused on the quality of training, according to studies^(11,21,27)^ and the objectives of the Global Strategy for 2025–2034^(12)^. Research points to weaknesses in the training of nurses in PICS^(27,28)^, as well as the need for minimum guidelines regarding the training of nursing professionals in PICS, pointing out that standardization qualifies and improves clinical safety^(7,27)^, an aspect contemplated in the objective of the experience report, which was based on training structured according to an unprecedented guideline developed in a thesis.

These aspects corroborate international purposes, given that among the challenges mentioned in the Global Strategy for TCIM 2025–2034, approved in May 2025, item 2.2, is the need to define minimum training requirements^(12)^. In other words, concern about the quality of training and, consequently, curricula and their minimum requirements is a growing issue.

Authors argue that the compilation/creation of reference documents for training and practices in Traditional, Complementary, and Integrative Healthcare (TCIH) should be a collaborative process between the professional organizations involved, educational institutions, and the WHO/regulator. Professionals can provide information on the educational outcomes required for the profession, help gather data on safety, efficacy, and economics, and play a significant role in regulating practices^(29)^.

There is a lack of studies addressing the reality of Portugal in the specific context of auriculotherapy/auricular acupuncture. A recent survey aimed to identify the attitudes, knowledge, and perspectives of 4,334 Portuguese physicians regarding NCMs, with almost half (45.5%) feeling uncomfortable talking about, discussing, and explaining NCMs^(30)^. The authors emphasize the need to include more content on the effectiveness and safety of CNT in medical training curricula, given that knowledge is a way to provide safe guidance.

It is understood that these aspects can be transposed to the reality of Portuguese nursing, which is directly involved in care. This reinforces the pioneering nature of this experience report, as it develops the first training based on a guideline for training nurses in auriculotherapy/auricular acupuncture that is unprecedented and scientifically qualified.

## CONCLUSION

The pedagogical experience reported is unprecedented and innovative, given that it was based on a guideline proposal structured at the National Workshop in Brazil.

It is hoped that this experience report will promote the movement to regulate auriculotherapy/auricular acupuncture as an intervention to be developed in services as a competence of nurses who have completed the minimum training, based on the Brazilian model.

## DATA AVAILABILITY

The entire dataset supporting the results of this study is available upon request to the corresponding author.
