# Supplementary material for: Training nurses in auriculotherapy/auricular acupuncture: possible paths for Portugal
Source: Rev Esc Enferm USP. 2026 Jan 26;60:e20250397. doi: 10.1590/1980-220X-REEUSP-2025-0397en (PMC12834389; doi:10.1590/1980-220X-REEUSP-2025-0397en)
Supplement: Translation

# Translation: Formação de enfermeiras(os) em auriculoterapia/acupuntura auricular: percursos possíveis para Portugal

**DOI:** 10.1590/1980-220X-REEUSP-2025-0397pt

**Authors:** Daiana Cristina Wickert, Diéssica Roggia Piexak, Daniela Dallegrave, Tereza Maria Mendes Diniz de Andrade Barroso, Maria Denise Schimith

**Affiliations:** 1 Universidade Federal de Santa Maria, Programa de Pós-Graduação em Enfermagem, Santa Maria, RS, Brasil.; 2 Universidade Federal do Rio Grande, Programa de Pós-Graduação em Enfermagem, Rio Grande, RS, Brasil.; 3 Universidade Federal do Rio Grande do Sul, Escola de Enfermagem e de Saúde Coletiva, Departamento de Assistência e Orientação Profissional, Porto Alegre, RS, Brasil.; 4 Universidade de Coimbra, Escola Superior de Enfermagem, Unidade de Investigação em Ciências da Saúde-Enfermagem, Coimbra, Portugal.

**Keywords:** Auriculoterapia, Acupuntura Auricular, Enfermagem, Ensino, Saúde Pública

## Abstract

**Objetivo:**

Relatar a experiência do desenvolvimento de uma formação em auriculoterapia/acupuntura auricular para enfermeiras(os) em Portugal.

**Método:**

Relato de experiência descritivo, utilizando o referencial metodológico proposto por Donabedian e análise descritiva informada e dialogada. A formação realizada faz parte de uma tese.

**Resultados:**

A experiência ocorreu de janeiro a fevereiro de 2025, durante mobilidade acadêmica na Escola Superior de Enfermagem de Coimbra. Trata-se de uma formação coletiva, desenvolvida com 19 enfermeiras(os) atuantes em Portugal e sem formação prévia em auriculoterapia, convidados de forma intencional. O curso foi adaptado seguindo a proposta de diretriz para formação de enfermeiras(os) em auriculoterapia/acupuntura auricular tendo seus objetivos educacionais pautados na Taxonomia de Bloom. O curso teve 75 horas teóricas on-line. Foi testada a carga horária prática, variando de cinco a 20 horas.

**Conclusão:**

A experiência é inédita e inovadora e poderá subsidiar regulamentação da formação em auriculoterapia/acupuntura auricular como intervenção de enfermeiras(os), tendo como base o modelo brasileiro.

## INTRODUÇÃO

As Medicinas Tradicionais, Complementares e Integrativas (MTCI), como são designadas pela Organização Mundial da Saúde (OMS), são práticas, produtos e saberes utilizados por diferentes povos e culturas do mundo. Internacionalmente as MTCI recebem distintas nomenclaturas, uma delas é Medicina Complementar e Alternativa (MAC), no inglês *Complementary and Alternative Medicine* (CAM)^(1)^.

No Brasil, as MTCI ou CAM são denominadas de Práticas Integrativas e Complementares em Saúde (PICS) que em 2006, foram consolidadas por meio da Política Nacional de Práticas Integrativas e Complementares (PNPIC) no Sistema Único de Saúde (SUS), um importante marco para o avanço das 29 práticas reconhecidas no SUS^(2)^, das quais a acupuntura auricular está contemplada no escopo de práticas da acupuntura/Medicina Tradicional Chinesa (MTC)^(3)^. Devido a diversidade de nomenclaturas que variam de acordo com a vertente ou escola, utilizar-se-á no presente artigo o termo auriculoterapia/acupuntura auricular para se referir a terapia auricular, na tentativa de garantir maior abrangência.

Em Portugal, as MTCI são conhecidas comumente como terapias ou intervenções não farmacológicas (INF)^(4)^. Usa-se também a nomenclatura Terapêuticas Não Convencionais (TNC) definidas como “Terapêuticas que partem de uma base filosófica diferente da medicina convencional e aplicam processos específicos de diagnóstico e terapêuticas próprias”. Em 2003, foram reconhecidas no país a Acupuntura, Fitoterapia, Homeopatia, Naturopatia, Osteopatia e Quiropraxia (Lei n. 45/2003), e, em 2013, a MTC (Lei n. 71/2013)^(5)^. Ainda, o termo Terapias Complementares e Alternativas (TCA) também é utilizado no contexto da enfermagem portuguesa^(6)^.

A enfermagem, que é caracterizada por seu trabalho voltado ao cuidado, pode utilizar a auriculoterapia como uma ferramenta promotora de autonomia, ao incluir a intervenção na sua prática assistencial^(7)^. Recente estudo metodológico construiu e validou o conteúdo dos componentes da intervenção de enfermagem em auriculoterapia, contribuindo assim para uma possível inclusão da auriculoterapia como intervenção de enfermagem na *Nursing Interventions Classification* (NIC), o que demonstra um avanço quanto à linguagem padronizada na prática clínica^(8)^.

Em termos de regulamentação, no Brasil, destaca-se a resolução do Conselho Federal de Enfermagem (COFEN), nº 739, de 05 de fevereiro de 2024, que reconheceu a auriculoterapia como capacitação por meio de cursos livres, recomendando carga horária mínima de 80 horas^(9)^, porém, sem maiores direcionamentos. A resolução apresenta a acupuntura separada da auriculoterapia, como especialização de, no mínimo, 360h. Já em Portugal, a acupuntura é uma licenciatura^(10)^.

Em Portugal, apesar de não existir uma regulamentação específica da Ordem dos Enfermeiros para o uso da auriculoterapia, documentos institucionais vêm mostrando o uso de práticas com o mesmo princípio no contexto de atuação do enfermeiro. Um Relatório de Mestrado em Enfermagem de Saúde Materna e Obstetrícia da Escola Superior de Enfermagem de Lisboa, que utiliza a acupressão como intervenção de enfermagem, aponta que a Mesa do Colégio da Especialidade de Saúde Materna e Obstétrica (MCEESMO) preconiza o uso de métodos não farmacológicos para alívio da dor durante o trabalho de parto e, entre as técnicas recomendadas incluem-se a acupressão. A autora descreve que a acupressão se baseia nos mesmos princípios da acupuntura, mas que as agulhas são substituídas por aplicação de pressão, o que é um benefício na medida em a torna uma técnica não invasiva sem necessidade de equipamento específico^(6)^.

Dessa forma, as autoras reforçam o caráter independente da formação em auriculoterapia/acupuntura auricular oferecida como curso livre ou qualificação profissional. As autoras compreendem que, por estudar apenas o microssistema da orelha, a auriculoterapia não é uma prática dependente da acupuntura^(11)^, ou seja, não é necessária a formação em acupuntura sistêmica para aprender a auriculoterapia/acupuntura auricular.

Frequentemente o trabalho da enfermagem está associado com a lógica das MTCI. Porém, hoje, não existe uma diretriz com parâmetros mínimos para a formação de enfermeiras(os) em auriculoterapia/acupuntura auricular.

Como produto de inquérito nacional sobre formação e atuação de enfermeiros em PICS no Brasil, por meio de uma tese de doutorado aninhada ao estudo, foi desenvolvida uma diretriz de formação para enfermeiras(os) em auriculoterapia/acupuntura auricular (ISBN nº 978-65-01-86051-0), e que foi utilizada como base para adaptação do curso de formação que sustenta o presente relato de experiência.

Nesse sentido, apresentam-se as justificativas deste relato de experiência:

A qualificação da formação de enfermeiras(os) reflete a assistência em saúde para um grande contingente de pessoas, dado que o número mais expressivo de trabalhadores da saúde é o da enfermagem. A enfermagem está presente em todo o mundo, nos lugares mais diversos.

O estudo propõe a reunião de elementos para embasar a formação profissional de enfermeiras(os) em auriculoterapia/acupuntura auricular. Ao mesmo tempo, essa sistematização pode abranger ou ser aplicada a outras áreas muito semelhantes ou próximas, como é o caso de outras MTCI ou outras profissões da saúde.

A auriculoterapia/acupuntura auricular é realizada por enfermeiras(os) de forma expressiva. A segurança do paciente, a adoção de práticas baseadas em evidências e a eficácia no cuidado de enfermagem são resultados esperados com a melhora da formação profissional, um dos objetivos da Estratégia Global para MTCI 2025–2034^(12)^.

Assim, objetiva-se relatar a experiência do desenvolvimento de uma formação em auriculoterapia para enfermeiras(os) ocorrida em Coimbra – Portugal.

## MÉTODO

### Desenho do Estudo

Trata-se de um relato de experiência que apresenta uma formação em auriculoterapia/acupuntura auricular desenvolvida com enfermeiras(os) portuguesas(os), com caráter descritivo. Este tipo de estudo descreve uma experiência inovadora sobre uma formação amplamente difundida no contexto da enfermagem brasileira, porém, ainda escassa em Portugal.

O eixo da experiência é a pesquisa, tendo em vista que a formação realizada faz parte de um projeto de tese que objetiva desenvolver uma diretriz para formação em auriculoterapia/acupuntura auricular, tendo por base os *benchmarks* da OMS. Espera-se que a experiência possa fomentar novos projetos que abordem a formação.

Destaca-se que inexiste um *guideline* com recomendações para a escrita de relatos de experiência, no entanto, optou-se por seguir os pressupostos para a elaboração de relato de experiência como conhecimento científico proposto por Mussi et al.^(13)^.

### Cenário

A experiência ocorreu em Coimbra, Portugal, durante formação avançada (mobilidade acadêmica) na Escola Superior de Enfermagem de Coimbra (ESEnfC). Coimbra é uma cidade portuguesa localizada na região central do país.

O local de realização foi a Ordem dos Enfermeiros (OE) de Coimbra, em uma sala cedida para desenvolver a formação. A OE, criada em 1998, é a associação pública profissional que incorpora todos os profissionais de Enfermagem que trabalham em Portugal, deliberando sobre a regulamentação dos enfermeiros e enfermeiros especialistas. A OE de Coimbra situa-se na mesma rua do Polo A da ESEnfC. Trata-se de um prédio acessível, em que foi fornecido o aporte estrutural e tecnológico necessário para o bom andamento da formação.

A ESEnfC, nasceu em 2006, da fusão da Escola Superior de Enfermagem Dr. Ângelo da Fonseca (fundada em 1881) e da Escola Superior de Enfermagem de Bissaya Barreto (fundada em 1971). A ESenfC possui três polos e uma Unidade de Investigação em Ciências da Saúde: Enfermagem (UICISA: E), a qual contempla um curso de licenciatura em Enfermagem, 11 cursos de mestrado, seis pós-graduações e um curso de doutorado, totalizando mais de 1900 estudantes inscritos na instituição. Está atualmente passando por um processo de integração com a Universidade de Coimbra e estará formalmente integrada até janeiro de 2026, conforme Decreto-Lei n.º 83/2024 (https://files.diariodarepublica.pt/1s/2024/10/21200/0001700023.pdf).

### Descrição do Processo

Os resultados descritos neste estudo são fruto do planejamento e condução de um curso de formação em auriculoterapia com enfermeiros(as) de Portugal. Especificamente a condução do curso ocorreu no período de janeiro a fevereiro de 2025.

### Análise de Dados

Para descrição da experiência foi utilizado o referencial metodológico proposto por Donabedian^(14)^, dividido em:

(1) Estrutura: buscou-se avaliar os recursos disponíveis como a qualificação das professoras, a infraestrutura da OE e os materiais didáticos utilizados.
(2) Processo: condução da formação, métodos de ensino e atividades práticas.
(3) Resultado: avaliar os efeitos da formação, a capacidade de aplicação da auriculoterapia e os impactos na sociedade.

A escolha do referencial se deu por permitir uma visão sistêmica e organizada de todos os aspectos que cerceiam a experiência descrita. Mesmo não se tratando de uma avaliação em saúde, a tríade de Donabedian mostrou-se adequada para descrever a formação de enfermeiras(os) em auriculoterapia/acupuntura auricular.

Como forma de análise, será realizada uma descrição informativa e dialogada conforme Mussi et al.^(13)^, tendo em vista se tratar de uma formação inédita vinculada a uma etapa de pesquisa.

### Aspectos Éticos

Este relato de experiência derivou das atividades desenvolvidas durante uma mobilidade acadêmica que contempla uma etapa de uma pesquisa de doutorado em enfermagem, aprovado pela Comissão de Ética da Sociedade Portuguesa de Enfermagem de Saúde Mental (CE-ASPESM) e pelo Comitê de Ética em Pesquisa da Universidade Federal do Rio Grande do Sul (UFRGS), sob Certificado de Apresentação para Apreciação Ética (CAAE) nº 43306921.6.0000.5347.

## RESULTADOS

### Estrutura Para Realização da Formação

A parceria entre docente da ESEnfC e as pesquisadoras brasileiras é oriunda de projetos anteriores, bem como, o interesse mútuo em levar a formação em auriculoterapia/acupuntura auricular para enfermeiras(os) de Portugal, tendo como base a experiência brasileira nesse aspecto.

Desta forma, o planejamento para viabilizar a realização da formação iniciou com a inscrição de um projeto de doutorado para concorrer ao edital da Fundação de Amparo à Pesquisa do Estado do Rio Grande do Sul (FAPERGS) número 04/2024- Auxílio emergencial para mobilidade de estudantes de doutorado em função da emergência climática ocorrida no Rio Grande do Sul (RS), lançado em 21 de junho de 2024, o qual as autoras concorreram e foram contempladas. O objetivo do edital era apoiar financeiramente a mobilidade de estudantes de doutorado regularmente matriculados em programas de pós- graduação de instituições com sede no estado do RS, Brasil. A mobilidade acadêmica, no caso deste relato, compreendeu um período de realização de atividades diretamente relacionadas ao projeto na ESEnfC em Portugal.

A base científica e metodológica para a proposta de formação desenvolvida em Portugal, além da prática clínica, advém das análises e discussões oriundas da Pesquisa EnfPICS (Inquérito nacional sobre o perfil educacional e profissional de enfermeiros de saúde integrativa e práticas tradicionais), desenvolvida por pesquisadoras brasileiras que vem aprofundando o conhecimento sobre o perfil e a formação de enfermeiros em auriculoterapia. No total, nove artigos e um capítulo de livro foram publicados. Ainda, foram realizadas palestras em eventos da área, como o IV CONGREPICS, a IV Jornada de Práticas Integrativas e Complementares em Saúde da Universidade de São Paulo (USP), 3º Congresso Mundial de Medicinas Tradicionais, Complementares e Integrativas, o 16º Congresso Internacional da Rede Unida no qual foi realizada a I Oficina Nacional para construção de diretrizes de formação para enfermeiras(os) em PICS: auriculoterapia, dando origem ao esboço da proposta de diretriz para formação de enfermeiras(os) em auriculoterapia/acupuntura auricular que guiou a realização da formação que será relatada.

Os materiais didáticos utilizados foram oriundos de uma adaptação do curso de extensão promovido pelo Programa de Extensão #SUStentaPICS (Escola de Enfermagem e de Saúde Coletiva – UFRGS), em parceria com a Secretaria de Estado da Saúde do RS (Área Técnica de PICS) e com o apoio da ABENAH, intitulado “Qualificação profissional em PICS: auriculoterapia”. O curso foi adaptado seguindo a proposta de diretriz para formação de enfermeiras(os) em auriculoterapia/acupuntura auricular.

A adaptação do curso passou por aprovação da Comissão de Extensão (COMEX) da Escola de Enfermagem e de Saúde Coletiva da UFRGS e da Câmara de Extensão (CAMEX) da UFRGS. O curso teve a etapa teórica on-line, via Moodle da UFRGS e a etapa prática presencial na OE em Coimbra, Portugal. Os materiais para aula prática foram doados e entregues aos 19 enfermeiros participantes do curso.

O curso foi ministrado por duas profissionais com formações em cursos livres em auriculoterapia. Uma delas enfermeira da APS há três anos no Brasil, doutora em enfermagem, associada na Associação Brasileira de Enfermeiros Acupunturistas e Enfermeiros em Práticas Integrativas (ABENAH) e no Consórcio Acadêmico Brasileiro de Saúde Integrativa (CABSIN), e a outra, doutora em enfermagem, docente do magistério superior na Universidade Federal de Rio Grande (FURG) há dez anos, associada no CABSIN e no Grupo de Trabalho Racionalidades Médicas e Práticas Integrativas e Complementares da Associação Brasileira de Saúde Coletiva. As profissionais possuem experiência clínica diária no uso da auriculoterapia/acupuntura auricular na prática de enfermagem e vêm desenvolvendo pesquisas inéditas na área, como pesquisadoras do EnfPICS.

### Processo de Desenvolvimento da Formação

Os processos realizados foram delineados considerando as etapas descritas no planejamento da formação a partir de oito objetivos de aprendizagem definidos à luz da Taxonomia de Bloom:

OBJETIVO 1 – Desenvolver e incentivar ações favoráveis a Educação Permanente em Saúde (EPS), com o intuito de garantir a formação e atualização da equipe de enfermagem no âmbito da auriculoterapia/acupuntura auricular e seus derivados;

OBJETIVO 2 – Utilizar os conceitos e visão de ser humano integral e de acolhimento como modelo de atendimento no oferecimento da auriculoterapia/acupuntura auricular e seus derivados como intervenção de enfermagem;

OBJETIVO 3 – Realizar a consulta e o cuidado de enfermagem com base nos princípios que regem a prática da auriculoterapia/acupuntura auricular e seus derivados;

OBJETIVO 4 – Indicar, prescrever e implementar a auriculoterapia/acupuntura auricular e seus derivados na oferta de cuidados de enfermagem, em qualquer serviço de saúde, nos âmbitos privado e público, reconhecendo-a como uma estratégia que contribui com a promoção da saúde global do indivíduo, associada aos demais cuidados e às racionalidades médicas;

OBJETIVO 5 – Realizar diagnósticos de enfermagem a partir das taxonomias reconhecidas pela categoria, estabelecer fatores relacionados e características definidoras e inserir a auriculoterapia/acupuntura auricular e seus derivados como intervenção de enfermagem (a acupuntura auricular e auriculoterapia não são intervenções de enfermagem reconhecidas pelas taxonomias, sendo esta uma lacuna a ser preenchida. Atualmente, intervenções relacionadas são: Estimulação cutânea e Acupressão);

OBJETIVO 6 – Aplicar auriculoterapia/acupuntura auricular e seus derivados com segurança, considerando os protocolos sanitários, com os diferentes estímulos de dispositivos invasivos (agulhas e derivados) e não invasivos (esferas, sementes, laser, acupressão e derivados);

OBJETIVO 7 – Promover o desenvolvimento do conhecimento científico de enfermagem, atuando e coordenando atividades de ensino/docência, pesquisa e formações em auriculoterapia/acupuntura auricular e seus derivados;

OBJETIVO 8 – Coordenar, planejar, organizar e orientar a equipe de enfermagem na implementação da auriculoterapia/acupuntura auricular e seus derivados na assistência de enfermagem;

Sendo assim, considerando os objetivos de aprendizagem o curso foi divido em seis núcleos de aprendizagem (cinco teóricos e um prático):

Núcleo de aprendizagem 1 (15h) – Auriculoterapia: apresentação da formação em terapia auricular. Apresentação do curso; Experiências de práticas integrativas e complementares nos EUA, América latina, países do oriente e Europa; Introdução à Auriculoterapia.

Núcleo de aprendizagem 2 (15h) – Auriculoterapia: Bases teóricas de acordo com a reflexologia: Estruturas anatômicas e pontos auriculares; Métodos de avaliação na auriculoterapia; Métodos de tratamento.

Núcleo de aprendizagem 3 (15h) – Auriculoterapia: Bases teóricas da medicina tradicional chinesa (MTC): Racionalidades Médicas; Auriculoterapia de acordo com os fundamentos da MTC.

Núcleo de aprendizagem 4 (15h) – Auriculoterapia: Bases teóricas segundo a biomedicina: Neurofisiologia; evidências biomédicas e potenciais efeitos adversos; linhas vermelhas.

Núcleo de aprendizagem 5 (15h) – Auriculoterapia nos cuidados de saúde primários: Cuidados de Saúde Primários e Auriculoterapia; uso da Auriculoterapia na rotina de atendimento dos cuidados de saúde primários; educação permanente e Auriculoterapia nos cuidados de saúde primários, e ainda nas consultas da dor na área da oncologia.

Núcleo de aprendizagem 6 (5h a 20h) – Aulas práticas: Inspeção e palpação do pavilhão auricular; fixação da semente no pavilhão auricular; acompanhamento clínico.

Trata-se de uma formação coletiva, desenvolvida com enfermeiras(os) atuantes em Portugal e sem formação prévia em auriculoterapia/acupuntura auricular, sendo esses os critérios de inclusão. A turma foi composta por 19 enfermeiras(os) portuguesas(es). Foram convidados de forma intencional por professora da ESEnfC a participar da formação. Os participantes eram enfermeiras(os) já conhecidas(os) pela professora por terem realizado especialização, mestrado ou doutorado na ESEnfC, ou ainda, por atuarem em serviços em que a mesma possui vínculo devido às aulas e projetos da Universidade. Foi realizada uma lista de interesse contendo nome, e-mail e telefone dos possíveis profissionais participantes.

Em um primeiro momento, no dia 03 de janeiro de 2025, foi criado um grupo de mensagens instantâneas por telefone móvel com os participantes, no intuito de facilitar a comunicação e sanar as dúvidas referentes ao cadastro no Moodle e no Portal da Extensão da Pró-Reitoria de Extensão (PROREXT) da UFRGS para iniciar as aulas teóricas. Além do grupo de mensagens instantâneas, as comunicações ocorreram também via e-mail.

Na etapa teórica, os materiais utilizados foram disponibilizados via Moodle da UFRGS. Os módulos foram liberados de acordo com o desenvolvimento de cada estudante, conforme conclusão das leituras e resolução das questões para avaliação do aprendizado. Os materiais consistiram em apostilas, artigos científicos, link direcionando para o mapa de evidências da Biblioteca Virtual em Saúde da MTCI, recomendações clínicas em auriculoterapia baseadas em evidências e vídeos de apresentação e demonstrando os materiais necessários para a aula prática. O diálogo foi constante e as dúvidas foram sanadas de forma coletiva e individualizada, conforme necessidade dos enfermeiros, via mensagem ou e-mail.

Na etapa prática, o espaço do auditório da OE foi utilizado. As mesas e cadeiras foram dispostas em círculo objetivando o diálogo constante entre os enfermeiros. Foram organizados quatro dias de aula prática, conforme período de realização da mobilidade acadêmica, sendo eles: 30 de janeiro, 13, 20 e 27 de fevereiro, das 13h às 18h, totalizando 20 horas de prática. Devido à liberação dos serviços de saúde, nem todos os enfermeiros estavam presentes em todos os dias de aula prática, sendo necessário um mínimo de 5h para certificação.

No dia 30 de janeiro ocorreu a primeira aula prática, a qual consistiu na apresentação pessoal e profissional, resolução de dúvidas sobre os módulos teóricos vistos até o momento (haviam concluído até o módulo 3) e a familiarização com os materiais e montagem das placas com sementes. Nesse momento, cada enfermeiro manifestou as datas que poderiam estar presentes na prática, sendo organizado o cronograma das atividades conforme disponibilidade do grupo, conforme Tabela 1.

**Tabela 1:** Cronograma das aulas práticas supervisionadas e número de participantes – Coimbra, Portugal, 2025.

Data da aula prática	Número de enfermeiros participantes
30 de janeiro	18 enfermeiros
13 de fevereiro	13 enfermeiros
20 de fevereiro	14 enfermeiros
27 de fevereiro	5 enfermeiros

Com os enfermeiros que realizaram 15 horas de aulas práticas, a formação foi pautada na biomedicina, reflexologia e MTC. Já com a turma que realizou 10 horas de prática, a abordagem foi voltada a biomedicina e reflexologia. Com base na experiência brasileira, as autoras compreendem que é possível aplicar a auriculoterapia pautada apenas na biomedicina e na reflexologia com qualidade e segurança com 5h de prática supervisionada devido à base de formação da graduação em enfermagem. No caso da MTC, 5h são consideradas mínimas e introdutórias, sendo necessário o aprofundamento com a prática profissional. De acordo com a experiência com as(os) enfermeiras(os) de Portugal, 10h foi avaliado como o mínimo necessário.

Como estratégia de ensino para a localização dos pontos auriculares, foi utilizado modelo de orelha de silicone e alfinetes, localização *in loco* na orelha do colega e por meio do uso de desenho da orelha e mapa auricular, conforme Figura 1.

**Figura 1:** Identificação de pontos auriculares em modelo de silicone, desenho da orelha e pessoa – Coimbra, Portugal, 2025.

 Sendo assim, a primeira parte da prática consistiu na localização correta dos principais pontos auriculares. Após, ao enfocar na auriculoterapia e derivados segundo a reflexologia e a biomedicina, foi apresentada a inervação da orelha (nervo aurículo temporal, vago e auricular maior) e o mecanismo de ação. No intuito de apreensão da técnica de inspeção auricular, a turma foi dividida em duplas ou trios, identificando mudanças de coloração, morfologia e vascularização uns nos outros, utilizando focos de luz. Em seguida, os pares treinaram a palpação auricular e identificação dos pontos.

Após, foi apresentado caso clínico para simular atendimento, no qual tiveram que localizar os pontos segundo a biomedicina e reflexologia e aplicar os pontos utilizando sementes nos colegas.

Ao aprofundar a auriculoterapia/acupuntura auricular segundo a MTC, foi utilizado como guia um livro^(15)^, abordando os diferentes materiais e seus estímulos: harmonização (sementes, cristais e pastilhas de silício), tonificação (agulhas 1,0 a 1,5 mm, agulhas superficiais e esferas de ouro) e sedação (agulhas de 1,8 a 2,0 mm, agulhas com profundidade e esferas de prata ou inox). Em seguida, foi retomada a teoria dos cinco elementos (vista na etapa teórica) e trabalhadas duas formas de tratamento segundo a MTC: (1) Tratamento do elemento/órgão choque e (2) Lei dos Ciclos de Geração e Dominância. Por fim, foram realizadas simulações com casos clínicos e protocolos de tratamento no intuito de praticar as três vertentes estudadas.

### Resultado da Formação

Após a formação, sob a forma de roda de conversa, avaliou-se a formação ofertada e o quanto as e os enfermeiras(os) se sentiam aptas(os) para aplicar a auriculoterapia/acupuntura auricular. Os *feedbacks* foram positivos quanto à qualidade da condução das aulas e aprendizados. Como aspectos negativos, foram mencionados a necessidade de uma formação com mais carga horária para utilização da terapia auricular segundo a MTC. Ainda, foi debatida a forma de regulamentação adotada pelo COFEN no Brasil e os caminhos para uma possível regulamentação da prática profissional pela OE em Portugal.

O grupo de mensagens instantâneas foi mantido e frequentemente ocorrem discussões de casos, dúvidas e *feedbacks*. Como impacto da formação, é esperado que ocorra uma difusão da auriculoterapia/acupuntura auricular em Portugal, fomentando assim novas turmas e novos horizontes no que se refere à mesma como intervenção no cuidado de enfermagem.

## DISCUSSÃO

O termo acupuntura auricular faz parte do escopo de práticas da MTC. As evidências não possuem um consenso acerca da origem da terapia auricular, que é utilizada há milhares de anos em diversos países, no entanto, o maior desenvolvimento da técnica e os primeiros documentos registrados são da China^(3)^. A auriculoterapia, de acordo com o manuscrito recente de Raphael Nogier, é um termo derivado dos estudos de seu pai, Paul Nogier, médico francês^(16)^. O termo auriculoterapia, no Brasil, é comumente utilizado para se referir a qualquer vertente da prática. Vale ressaltar que os estudos de Paul Nogier impulsionaram o reconhecimento da auriculoterapia no contexto internacional^(16)^. Tendo em vista a discussão na comunidade científica acerca da terminologia, a opção pelo uso do termo auriculoterapia/acupuntura auricular objetivou contemplar as diferentes escolas e vertentes da prática, todas com sua importância e relevância científica.

A MTC é uma racionalidade médica composta de uma “complexa teia de interações, com alguns momentos de afastamento, outros de aproximação, entre várias escolas e tradições que surgiram ao longo da história”^(17:104)^, e que hoje é praticada no mundo todo. Atualmente, a MTC está subdividida em especialidades (semelhante à tendência da biomedicina), fato que pode ter influência da ciência cartesiana. No entanto, é preciso lembrar que na sua origem, a terapêutica da MTC combinava modalidades de tratamento, como por exemplo: acupuntura, massagem, farmacopeia, dietoterapia e exercícios terapêuticos^(17)^.

O documento mais importante no contexto mundial sobre a formação em acupuntura é o *WHO Benchmarks for the training of acupuncture*, desenvolvido pela OMS em parceria com mais de 50 especialistas do mundo todo. O mesmo estabelece categorias de treinamento em acupuntura, níveis de dificuldade dos conteúdos e uma grade curricular dos módulos de aprendizagem, que possui a intersecção de conteúdos da MTC diante das disciplinas básicas da área da saúde^(18)^.

Não obstante, o destaque nesta discussão é o reconhecimento do caráter multiprofissional da acupuntura, em conformidade com a OMS^(19)^, tendo em vista que o documento faz uma diferenciação no currículo básico para profissões biomédicas, pessoas que praticam medicinas tradicionais e pessoas sem formação biomédica^(18)^. Considerando a acupuntura auricular como uma prática que está contemplada no escopo da acupuntura, mas que a formação pode ser realizada de forma independente, por meio de cursos livres (independentemente da formação em acupuntura), reforça também seu caráter multiprofissional. Evidência indica que enfermeiros que trabalham na área da MTC levaram à melhoria do estado de saúde dos pacientes, e que dentre as intervenções utilizadas está a auriculoterapia/acupuntura auricular^(20)^.

Pesquisa que avaliou o perfil de formação de profissionais de auriculoterapia no Brasil, identificou um grupo multidisciplinar de praticantes, sendo a maioria mulheres de meia-idade que atendiam em seus consultórios particulares e com formações de mais de 40 horas, no formato on-line^(21)^. Tal aspecto difere de pesquisa brasileira que avaliou apenas enfermeiros com formação em auriculoterapia, em que a maioria concentra seus atendimentos na APS^(11)^.

Nesse sentido, considerando o contexto português, o Inquérito Social Europeu, Rodada 7 (edição 2.0, 2014) identificou que 14,1% da população de Portugal utiliza alguma CAM para o cuidado. Quase uma década depois, o Inquérito Social Europeu, Rodada 11 (edição 2.0, 2023), mostrou os perfis típicos de usuários de CAM na Europa permaneceram estáveis: mulheres, grupos de meia-idade e pessoas com alto nível de escolaridade tendem a usar mais do que outros grupos^(22)^.

O exercício integrado das TNC juntamente com a medicina convencional no seio do Serviço Nacional de Saúde é sugerido na Lei de Bases da Saúde de 2019, na base 26, onde afirma que “O exercício das terapêuticas não convencionais é regulado pela lei, efetuado de modo integrado com as terapêuticas convencionais e de forma a garantir a proteção da saúde das pessoas e das comunidades, a qualidade assistencial e tendo por base a melhor evidência científica”^(23:63))^.

Recente revisão sistemática obteve como resultados que a utilidade percebida, definida como os benefícios percebidos de uma modalidade de MTCI no atendimento de necessidades ou objetivos de saúde específicos, é o fator mais influente no uso de MTCI, relatada em 79% dos estudos. Desta forma, os autores sugerem que ao integrar práticas de MTCI amplamente aceitas, as autoridades de saúde podem garantir o uso seguro, padronizado e baseado em evidências em ambientes médicos. Essa integração pode maximizar o engajamento e a satisfação do paciente, levando a melhores resultados de saúde^(24)^.

Com isso, analisar experiências internacionais como as do Brasil^(25)^, China e Estados Unidos da América (EUA), pode fomentar a implementação em outros contextos. Na China, a MTC é a principal prática, sendo que os esforços se voltam a traduzir as evidências e desenvolver diretrizes de prática clínica, tendo em vista que a MTC já foi adotada em todos os níveis de assistência à saúde. Nos EUA, o sistema de saúde é dominado pela medicina convencional, sendo assim, as intervenções de implementação voltam-se a facilitar o fornecimento de modalidades específicas de MTCI baseadas em evidências por meio de encaminhamentos de clínicos convencionais^(26)^.

No Brasil, tendo em vista as potencialidades e fragilidades na implantação da PNPIC, viabilizar a formação e qualificação dos profissionais em número adequado para atuar no SUS é uma necessidade. Neste sentido, autores afirmam que o interesse de profissionais em se capacitar e o número considerável de iniciativas de ensino e pesquisa nas universidades públicas podem catalisar este processo^(25)^.

Atualmente a preocupação das pesquisas volta-se também para a qualidade das formações, conforme estudos^(11,21,27)^ e objetivos da Estratégia Global para 2025–2034^(12)^. Pesquisas apontam a fragilidades nas formações de enfermeiros em PICS^(27,28)^, bem como, a necessidade de diretrizes mínimas com relação à formação de profissionais de enfermagem em PICS, apontando que a padronização qualifica e melhora a segurança clínica^(7,27)^, aspecto contemplado no objetivo do relato de experiência que pautou-se em uma formação estruturada em uma diretriz inédita elaborada em uma tese.

Tais aspectos corroboram com os anseios internacionais, tendo em vista que dentre os desafios citados na Estratégia Global para MTCI 2025–2034, aprovada em maio de 2025, no item 2.2, está a necessidade de definir requisitos mínimos de treinamento^(12)^. Ou seja, a preocupação com a qualidade das formações e consequentemente dos currículos e seus requisitos mínimos é um tema crescente.

Autores defendem que a compilação/criação de documentos de referência para a formação e práticas de *Traditional, complementary and integrative healthcare* (TCIH) (tradução nossa: Cuidados de saúde tradicionais, complementares e integrativos) deve ser um processo colaborativo entre as organizações profissionais envolvidas, as instituições educativas e a OMS/regulador. Os profissionais podem fornecer informações sobre os resultados educacionais necessários para a profissão, ajudar a reunir os dados de segurança, eficácia e economia e, desempenhar um papel significativo na regulação das práticas^(29)^.

Carecem estudos que abordem a realidade de Portugal no contexto específico da auriculoterapia/acupuntura auricular. Uma pesquisa recente objetivou identificar as atitudes, conhecimentos e perspectivas de 4334 médicos portugueses relativamente às TNC, sendo que quase metade (45,5%) não se sentem confortáveis para conversar, argumentar e explicar sobre as TNC^(30)^. Os autores realçam a necessidade de incluir nos currículos de formação médica mais conteúdos sobre a eficácia e segurança das TNC, tendo em vista que conhecer é uma forma de orientar com segurança.

Entende-se que tais aspectos podem ser transpostos para a realidade da enfermagem portuguesa, que está diretamente implicada no cuidado. O que reforça o pioneirismo do presente relato de experiência, ao desenvolver a primeira formação pautada em uma diretriz para formação de enfermeiras(os) em auriculoterapia/acupuntura auricular inédita e cientificamente qualificada.

## CONCLUSÃO

A experiência pedagógica relatada é inédita e inovadora, tendo em vista que foi pautada em uma proposta de diretriz estruturada na Oficina Nacional no Brasil.

Espera-se com o presente relato de experiência fomentar o movimento de regulamentação da auriculoterapia/acupuntura auricular como intervenção a ser desenvolvida nos serviços como competência das(os) enfermeiras(os) que realizaram a formação mínima, tendo como base o modelo brasileiro.

## DISPONIBILIDADE DE DADOS

Todo o conjunto de dados que dá suporte aos resultados deste estudo está disponível mediante solicitação ao autor correspondente.

# Translation: Formación de enfermeras en auriculoterapia/acupuntura auricular: posibles vías para Portugal

**DOI:** 10.1590/1980-220X-REEUSP-2025-0397es

**Authors:** Daiana Cristina Wickert, Diéssica Roggia Piexak, Daniela Dallegrave, Tereza Maria Mendes Diniz de Andrade Barroso, Maria Denise Schimith

**Affiliations:** 1 Universidade Federal de Santa Maria, Programa de Pós-Graduação em Enfermagem, Santa Maria, RS, Brasil.; 2 Universidade Federal do Rio Grande, Programa de Pós-Graduação em Enfermagem, Rio Grande, RS, Brasil.; 3 Universidade Federal do Rio Grande do Sul, Escola de Enfermagem e de Saúde Coletiva, Departamento de Assistência e Orientação Profissional, Porto Alegre, RS, Brasil.; 4 Universidade de Coimbra, Escola Superior de Enfermagem, Unidade de Investigação em Ciências da Saúde-Enfermagem, Coimbra, Portugal.

**Keywords:** Auriculoterapia, Acupuntura Auricular, Enfermería, Enseñanza, Salud Pública

## Abstract

**Objetivo:**

Relatar la experiencia del desarrollo de una formación en auriculoterapia/acupuntura auricular para enfermeros en Portugal.

**Método:**

Relato descriptivo de la experiencia, utilizando el marco metodológico propuesto por Donabedian y el análisis descriptivo informado y dialogado. La formación realizada forma parte de una tesis.

**Resultados:**

La experiencia tuvo lugar de enero a febrero de 2025, durante la movilidad académica en la Escuela Superior de Enfermería de Coimbra. Se trata de una formación colectiva, desarrollada con 19 enfermeros que trabajan en Portugal y sin formación previa en auriculoterapia, invitados de forma intencionada. El curso se adaptó siguiendo la propuesta de directrices para la formación de enfermeras en auriculoterapia/acupuntura auricular, con objetivos educativos basados en la taxonomía de Bloom. El curso contó con 75 horas teóricas en línea. Se probó la carga horaria práctica, que varió entre cinco y 20 horas.

**Conclusión:**

La experiencia es inédita e innovadora y podría servir de base para la regulación de la formación en auriculoterapia/acupuntura auricular como intervención de enfermeros, basándose en el modelo brasileño.

## INTRODUCCIÓN

Las Medicinas Tradicionales, Complementarias e Integrativas (MTCI), como las denomina la Organización Mundial de la Salud (OMS), son prácticas, productos y conocimientos utilizados por diferentes pueblos y culturas del mundo. A nivel internacional, las MTCI reciben diferentes denominaciones, una de ellas es Medicina Complementaria y Alternativa (MAC), en inglés *Complementary and Alternative Medicine* (CAM)^(1)^.

En Brasil, las MTCI o CAM se denominan Prácticas Integrativas y Complementarias en Salud (PICS), que en 2006 se consolidaron mediante la Política Nacional de Prácticas Integrativas y Complementarias (PNPIC) en el Sistema Único de Salud (SUS), un hito importante para el avance de las 29 prácticas reconocidas en el SUS^(2)^, entre las que se incluye la acupuntura auricular en el ámbito de las prácticas de acupuntura/Medicina Tradicional China (MTC)^(3)^. Debido a la diversidad de nomenclaturas que varían según la corriente o escuela, en el presente artículo se utilizará el término auriculoterapia/acupuntura auricular para referirse a la terapia auricular, en un intento de garantizar una mayor cobertura.

En Portugal, las MTCI se conocen comúnmente como terapias o intervenciones no farmacológicas (INF)^(4)^. También se utiliza la nomenclatura Terapias No Convencionales (TNC), definidas como “terapias que parten de una base filosófica diferente a la medicina convencional y aplican procesos específicos de diagnóstico y terapias propias”. En 2003, se reconocieron en el país la acupuntura, la fitoterapia, la homeopatía, la naturopatía, la osteopatía y la quiropráctica (Ley n.º 45/2003) y, en 2013, la MTC (Ley n.º 71/2013)^(5)^. Además, el término Terapias Complementarias y Alternativas (TCA) también se utiliza en el contexto de la enfermería portuguesa^(6)^.

La enfermería, que se caracteriza por su trabajo orientado al cuidado, puede utilizar la auriculoterapia como una herramienta que promueve la autonomía, al incluir la intervención en su práctica asistencial^(7)^. Un estudio metodológico reciente construyó y validó el contenido de los componentes de la intervención de enfermería en auriculoterapia, contribuyendo así a una posible inclusión de la auriculoterapia como intervención de enfermería en la Clasificación de Intervenciones de Enfermería (NIC), lo que demuestra un avance en cuanto al lenguaje estandarizado en la práctica clínica^(8)^.

En términos de regulación, en Brasil destaca la resolución del Consejo Federal de Enfermería (COFEN), n.º 739, de 5 de febrero de 2024, que reconoció la auriculoterapia como formación mediante cursos libres, recomendando una carga horaria mínima de 80 horas^(9)^, pero sin más orientaciones. La resolución presenta la acupuntura separada de la auriculoterapia, como una especialización de, al menos, 360 horas. En Portugal, la acupuntura es una licenciatura^(10)^.

En Portugal, aunque no existe una normativa específica del Colegio de Enfermeros para el uso de la auriculoterapia, los documentos institucionales muestran el uso de prácticas con el mismo principio en el contexto de la actuación del enfermero. Un informe de maestría en Enfermería de Salud Materna y Obstetricia de la Escuela Superior de Enfermería de Lisboa, que utiliza la acupresión como intervención de enfermería, señala que la Mesa del Colegio de la Especialidad de Salud Materna y Obstétrica (MCEESMO) recomienda el uso de métodos no farmacológicos para aliviar el dolor durante el trabajo de parto y, entre las técnicas recomendadas se incluye la acupresión. La autora describe que la acupresión se basa en los mismos principios que la acupuntura, pero que las agujas se sustituyen por la aplicación de presión, lo que supone una ventaja, ya que la convierte en una técnica no invasiva que no requiere equipo específico^(6)^.

De este modo, las autoras refuerzan el carácter independiente de la formación en auriculoterapia/acupuntura auricular ofrecida como curso libre o cualificación profesional. Las autoras entienden que, al estudiar solo el microsistema de la oreja, la auriculoterapia no es una práctica dependiente de la acupuntura^(11)^, es decir, no es necesaria la formación en acupuntura sistémica para aprender auriculoterapia/acupuntura auricular.

A menudo, el trabajo de enfermería se asocia con la lógica de la MTCI. Sin embargo, en la actualidad no existe una directriz con parámetros mínimos para la formación de enfermeros en auriculoterapia/acupuntura auricular.

Como resultado de una encuesta nacional sobre la formación y el desempeño de los enfermeros en PICS en Brasil, a través de una tesis doctoral vinculada al estudio, se desarrolló una directriz de formación para enfermeros en auriculoterapia/acupuntura auricular (ISBN nº 978-65-01-86051-0), que se utilizó como base para la adaptación del curso de formación que sustenta el presente informe de experiencia.

En este sentido, se presentan las justificaciones de este informe de experiencia:

La cualificación de la formación de enfermeros refleja la asistencia sanitaria para un gran contingente de personas, dado que el número más significativo de trabajadores sanitarios es el de enfermería. La enfermería está presente en todo el mundo, en los lugares más diversos.

El estudio propone reunir elementos para fundamentar la formación profesional de enfermeras en auriculoterapia/acupuntura auricular. Al mismo tiempo, esta sistematización puede abarcar o aplicarse a otras áreas muy similares o cercanas, como es el caso de otras MTCI u otras profesiones de la salud.

La auriculoterapia/acupuntura auricular es realizada por enfermeros de manera significativa. La seguridad del paciente, la adopción de prácticas basadas en la evidencia y la eficacia en la atención de enfermería son resultados esperados con la mejora de la formación profesional, uno de los objetivos de la Estrategia Global para la MTCI 2025–2034^(12)^.

Por lo tanto, el objetivo es relatar la experiencia del desarrollo de una formación en auriculoterapia para enfermeras que tuvo lugar en Coimbra (Portugal).

## MÉTODO

### Diseño del Estudio

Se trata de un informe de experiencia que presenta una formación en auriculoterapia/acupuntura auricular desarrollada con enfermeras portuguesas, de carácter descriptivo. Este tipo de estudio describe una experiencia innovadora sobre una formación ampliamente difundida en el contexto de la enfermería brasileña, pero aún escasa en Portugal.

El eje de la experiencia es la investigación, ya que la formación realizada forma parte de un proyecto de tesis que tiene como objetivo desarrollar una directriz para la formación en auriculoterapia/acupuntura auricular, basada en los *benchmarks* de la OMS. Se espera que la experiencia pueda fomentar nuevos proyectos que aborden la formación.

Cabe destacar que no existe una guía con recomendaciones para la redacción de informes de experiencia, sin embargo, se optó por seguir los supuestos para la elaboración de informes de experiencia como conocimiento científico propuestos por Mussi et al.^(13)^.

### Escenario

La experiencia tuvo lugar en Coimbra, Portugal, durante una formación avanzada (movilidad académica) en la Escuela Superior de Enfermería de Coimbra (ESEnfC). Coimbra es una ciudad portuguesa situada en la región central del país.

El lugar donde se llevó a cabo fue la Orden de Enfermeros (OE) de Coimbra, en una sala cedida para desarrollar la formación. La OE, creada en 1998, es la asociación profesional pública que agrupa a todos los profesionales de enfermería que trabajan en Portugal, y se encarga de deliberar sobre la regulación de los enfermeros y enfermeros especialistas. La OE de Coimbra se encuentra en la misma calle que el Polo A de la ESEnfC. Se trata de un edificio accesible, en el que se proporcionó el apoyo estructural y tecnológico necesario para el buen desarrollo de la formación.

La ESEnfC nació en 2006 de la fusión de la Escuela Superior de Enfermería Dr. Ângelo da Fonseca (fundada en 1881) y la Escuela Superior de Enfermería de Bissaya Barreto (fundada en 1971). La ESEnfC cuenta con tres centros y una Unidad de Investigación en Ciencias de la Salud: Enfermería (UICISA: E), que ofrece una licenciatura en Enfermería, 11 maestrías, seis posgrados y un doctorado, con un total de más de 1900 estudiantes matriculados en la institución. Actualmente se encuentra en proceso de integración con la Universidad de Coimbra y estará formalmente integrada en enero de 2026, de conformidad con el Decreto-Ley n.º 83/2024 (https://files.diariodarepublica.pt/1s/2024/10/21200/0001700023.pdf).

### Descripción del Proceso

Los resultados descritos en este estudio son el fruto de la planificación y realización de un curso de formación en auriculoterapia con enfermeros de Portugal. Concretamente, el curso se impartió entre enero y febrero de 2025.

### Análisis de Datos

Para describir la experiencia se utilizó el marco metodológico propuesto por Donabedian^(14)^, dividido en:

(1) Estructura: se buscó evaluar los recursos disponibles, como la cualificación de los profesores, la infraestructura de la OE y los materiales didácticos utilizados.
(2) Proceso: desarrollo de la formación, métodos de enseñanza y actividades prácticas.
(3) Resultado: evaluar los efectos de la formación, la capacidad de aplicación de la auriculoterapia y los impactos en la sociedad.

La elección del marco se debió a que permitía una visión sistémica y organizada de todos los aspectos que rodean la experiencia descrita. Aunque no se trata de una evaluación en salud, la tríada de Donabedian resultó adecuada para describir la formación de enfermeros en auriculoterapia/acupuntura auricular.

Como forma de análisis, se realizará una descripción informativa y dialogada según Mussi et al.^(13)^, teniendo en cuenta que se trata de una formación inédita vinculada a una etapa de investigación.

### Aspectos éticos

Este relato de experiencia se deriva de las actividades desarrolladas durante una movilidad académica que contempla una etapa de una investigación de doctorado en enfermería, aprobada por la Comisión de Ética de la Sociedad Portuguesa de Enfermería de Salud Mental (CE-ASPESM) y por el Comité de Ética en Investigación de la Universidad Federal de Rio Grande do Sul (UFRGS), bajo el Certificado de Presentación para Apreciación Ética (CAAE) n.º 43306921.6.0000.5347.

## RESULTADOS

### Estructura Para la Realización de la Formación

La colaboración entre el profesorado de la ESEnfC y las investigadoras brasileñas tiene su origen en proyectos anteriores, así como en el interés mutuo por llevar la formación en auriculoterapia/acupuntura auricular a las enfermeras de Portugal, basándose en la experiencia brasileña en este ámbito.

De este modo, la planificación para hacer posible la formación comenzó con la inscripción de un proyecto de doctorado para concurrir a la convocatoria de la Fundación de Apoyo a la Investigación del Estado de Rio Grande do Sul (FAPERGS) número 04/2024 – Ayuda de emergencia para la movilidad de estudiantes de doctorado debido a la emergencia climática ocurrida en Rio Grande do Sul (RS), lanzada el 21 de junio de 2024, a la que las autoras se presentaron y fueron seleccionadas. El objetivo de la convocatoria era apoyar financieramente la movilidad de los estudiantes de doctorado matriculados regularmente en programas de posgrado de instituciones con sede en el estado de RS, Brasil. La movilidad académica, en el caso de este informe, comprendió un período de realización de actividades directamente relacionadas con el proyecto en la ESEnfC en Portugal.

La base científica y metodológica de la propuesta de formación desarrollada en Portugal, además de la práctica clínica, proviene de los análisis y debates derivados de la investigación EnfPICS (Encuesta nacional sobre el perfil educativo y profesional de los enfermeros de salud integrativa y prácticas tradicionales), desarrollada por investigadoras brasileñas que han profundizado en el conocimiento sobre el perfil y la formación de los enfermeros en auriculoterapia. En total, se han publicado nueve artículos y un capítulo de libro. Además de haberse impartido conferencias en eventos del sector, como el IV CONGREPICS, la IV Jornada de Prácticas Integrativas y Complementarias en Salud de la Universidad de São Paulo (USP), 3.º Congreso Mundial de Medicinas Tradicionales, Complementarias e Integrativas, el 16º Congreso Internacional de la Red Unida, en el que se celebró el I Taller Nacional para la elaboración de directrices de formación de enfermeros en PICS: auriculoterapia, que dio lugar al borrador de la propuesta de directrices para la formación de enfermeros en auriculoterapia/acupuntura auricular que guió la realización de la formación que se describirá a continuación.

Los materiales didácticos utilizados procedían de una adaptación del curso de extensión promovido por el Programa de Extensión #SUStentaPICS (Escuela de Enfermería y Salud Colectiva – UFRGS), en colaboración con la Secretaría de Estado de Salud de RS (Área Técnica de PICS) y con el apoyo de ABENAH, titulado “Cualificación profesional en PICS: auriculoterapia”. El curso se adaptó siguiendo la propuesta de directrices para la formación de enfermeros en auriculoterapia/acupuntura auricular.

La adaptación del curso fue aprobada por la Comisión de Extensión (COMEX) de la Escuela de Enfermería y Salud Colectiva de la UFRGS y por la Cámara de Extensión (CAMEX) de la UFRGS. El curso contó con una etapa teórica en línea, a través de Moodle de la UFRGS, y una etapa práctica presencial en la OE en Coímbra, Portugal. Los materiales para la clase práctica fueron donados y entregados a los 19 enfermeros participantes en el curso.

El curso fue impartido por dos profesionales con formación en cursos libres de auriculoterapia. Una de ellas es enfermera de APS desde hace tres años en Brasil, doctora en enfermería, miembro de la Asociación Brasileña de Enfermeros Acupuntores y Enfermeros en Prácticas Integrativas (ABENAH) y del Consorcio Académico Brasileño de Salud Integrativa (CABSIN), y la otra, doctora en enfermería, profesora de educación superior en la Universidad Federal de Rio Grande (FURG) desde hace diez años, miembro del CABSIN y del Grupo de Trabajo de Racionalidades Médicas y Prácticas Integrativas y Complementarias de la Asociación Brasileña de Salud Colectiva. Las profesionales tienen experiencia clínica diaria en el uso de la auriculoterapia/acupuntura auricular en la práctica de la enfermería y han estado desarrollando investigaciones inéditas en el área, como investigadoras del EnfPICS.

### Proceso de Desarrollo de la Formación

Los procesos realizados se diseñaron teniendo en cuenta las etapas descritas en la planificación de la formación a partir de ocho objetivos de aprendizaje definidos a la luz de la taxonomía de Bloom:

OBJETIVO 1 – Desarrollar e incentivar acciones favorables a la Educación Permanente en Salud (EPS), con el fin de garantizar la formación y actualización del equipo de enfermería en el ámbito de la auriculoterapia/acupuntura auricular y sus derivados.

OBJETIVO 2 – Utilizar los conceptos y la visión del ser humano integral y de acogida como modelo de atención en la oferta de auriculoterapia/acupuntura auricular y sus derivados como intervención de enfermería.

OBJETIVO 3 – Realizar la consulta y la atención de enfermería basándose en los principios que rigen la práctica de la auriculoterapia/acupuntura auricular y sus derivados.

OBJETIVO 4 – Indicar, prescribir y implementar la auriculoterapia/acupuntura auricular y sus derivados en la prestación de cuidados de enfermería, en cualquier servicio de salud, en los ámbitos privado y público, reconociéndola como una estrategia que contribuye a la promoción de la salud global del individuo, asociada a otros cuidados y racionalidades médicas;

OBJETIVO 5 – Realizar diagnósticos de enfermería a partir de las taxonomías reconocidas por la categoría, establecer factores relacionados y características definitorias e insertar la auriculoterapia/acupuntura auricular y sus derivados como intervención de enfermería (la acupuntura auricular y la auriculoterapia no son intervenciones de enfermería reconocidas por las taxonomías, lo que constituye una laguna que debe colmarse). Actualmente, las intervenciones relacionadas son: estimulación cutánea y acupresión);

OBJETIVO 6 – Aplicar la auriculoterapia/acupuntura auricular y sus derivados de forma segura, teniendo en cuenta los protocolos sanitarios, con los diferentes estímulos de dispositivos invasivos (agujas y derivados) y no invasivos (esferas, semillas, láser, acupresión y derivados);

OBJETIVO 7 – Promover el desarrollo del conocimiento científico de enfermería, actuando y coordinando actividades de enseñanza/docencia, investigación y formación en auriculoterapia/acupuntura auricular y sus derivados.

OBJETIVO 8: – Coordinar, planificar, organizar y orientar al equipo de enfermería en la implementación de la auriculoterapia/acupuntura auricular y sus derivados en la asistencia de enfermería.

Así pues, teniendo en cuenta los objetivos de aprendizaje, el curso se dividió en seis núcleos de aprendizaje (cinco teóricos y uno práctico): Núcleo de aprendizaje 1 (15 h) – Auriculoterapia: presentación de la formación en terapia auricular. Presentación del curso; Experiencias de prácticas integrativas y complementarias en EE. UU., América Latina, países orientales y Europa; Introducción a la auriculoterapia. Núcleo de aprendizaje 2 (15 h) – Auriculoterapia: Fundamentos teóricos según la reflexología: Estructuras anatómicas y puntos auriculares; Métodos de evaluación en auriculoterapia; Métodos de tratamiento.

Núcleo de aprendizaje 3 (15 h) – Auriculoterapia: Fundamentos teóricos de la medicina tradicional china (MTC): Racionalidades médicas; Auriculoterapia según los fundamentos de la MTC.

Núcleo de aprendizaje 4 (15 h) – Auriculoterapia: Fundamentos teóricos según la biomedicina: Neurofisiología; evidencias biomédicas y posibles efectos adversos; líneas rojas.

Núcleo de aprendizaje 5 (15 h) – Auriculoterapia en la atención primaria: Atención primaria y auriculoterapia; uso de la auriculoterapia en la rutina de la atención primaria; formación continua y auriculoterapia en la atención primaria, así como en las consultas de dolor en el ámbito de la oncología.

Núcleo de aprendizaje 6 (5 a 20 h) – Clases prácticas: inspección y palpación del pabellón auricular; fijación de la semilla en el pabellón auricular; seguimiento clínico.

Se trata de una formación colectiva, desarrollada con enfermeras que trabajan en Portugal y sin formación previa en auriculoterapia/acupuntura auricular, siendo estos los criterios de inclusión. La clase estaba compuesta por 19 enfermeras portuguesas. Fueron invitadas intencionalmente por la profesora de la ESEnfC a participar en la formación. Los participantes eran enfermeros ya conocidos por la profesora por haber realizado una especialización, maestría o doctorado en la ESEnfC, o por trabajar en servicios con los que ella tiene vínculos debido a las clases y proyectos de la Universidad. Se elaboró una lista de interesados con el nombre, el correo electrónico y el teléfono de los posibles profesionales participantes.

En un primer momento, el 3 de enero de 2025, se creó un grupo de mensajes instantáneos por teléfono móvil con los participantes, con el fin de facilitar la comunicación y resolver las dudas relativas al registro en Moodle y en el Portal de Extensión de la Vicerrectoría de Extensión (PROREXT) de la UFRGS para iniciar las clases teóricas. Además del grupo de mensajes instantáneos, las comunicaciones también se realizaron por correo electrónico.

En la etapa teórica, los materiales utilizados se pusieron a disposición a través de Moodle de la UFRGS. Los módulos se liberaron de acuerdo con el desarrollo de cada estudiante, según la finalización de las lecturas y la resolución de las preguntas para la evaluación del aprendizaje. Los materiales consistieron en folletos, artículos científicos, enlaces que dirigían al mapa de evidencias de la Biblioteca Virtual en salud de la MTCI, recomendaciones clínicas en auriculoterapia basadas en evidencias y videos de presentación y demostración de los materiales necesarios para la clase práctica. El diálogo fue constante y las dudas se resolvieron de forma colectiva e individualizada, según las necesidades de los enfermeros, por mensaje o correo electrónico.

En la etapa práctica, se utilizó el espacio del auditorio de la OE. Las mesas y sillas se dispusieron en círculo con el objetivo de facilitar el diálogo constante entre los enfermeros. Se organizaron cuatro días de clases prácticas, de acuerdo con el período de realización de la movilidad académica, a saber: 30 de enero, 13, 20 y 27 de febrero, de 13:00 a 18:00, con un total de 20 horas de práctica. Debido a la liberación de los servicios de salud, no todos los enfermeros estuvieron presentes en todos los días de clases prácticas, siendo necesario un mínimo de 5 horas para la certificación.

El 30 de enero tuvo lugar la primera clase práctica, que consistió en la presentación personal y profesional, la resolución de dudas sobre los módulos teóricos vistos hasta el momento (se había completado hasta el módulo 3) y la familiarización con los materiales y el montaje de las placas con semillas. En ese momento, cada enfermero indicó las fechas en las que podría estar presente en la práctica, y se organizó el calendario de actividades según la disponibilidad del grupo, tal y como se muestra en la Tabla 1.

**Tabla 1:** Calendario de clases prácticas supervisadas y número de participantes – Coimbra, Portugal, 2025.

Fecha de la clase práctica	Número de enfermeros participantes
30 de enero	18 enfermeros
13 de febrero	13 enfermeros
20 de febrero	14 enfermeros
27 de febrero	5 enfermeros

Con los enfermeros que realizaron 15 horas de clases prácticas, la formación se basó en la biomedicina, la reflexología y la medicina tradicional china. En cuanto al grupo que realizó 10 horas de prácticas, el enfoque se centró en la biomedicina y la reflexología. Basándose en la experiencia brasileña, las autoras entienden que es posible aplicar la auriculoterapia basada únicamente en la biomedicina y la reflexología con calidad y seguridad con 5 horas de práctica supervisada, debido a la base formativa de la licenciatura en enfermería. En el caso de la MTC, 5 horas se consideran mínimas e introductorias, siendo necesario profundizar con la práctica profesional. Según la experiencia con las enfermeras de Portugal, se evaluó que 10 horas eran el mínimo necesario.

Como estrategia de enseñanza para la localización de los puntos auriculares, se utilizó un modelo de oreja de silicona y alfileres, la localización *in situ* en la oreja del compañero y mediante el uso de un dibujo de la oreja y un mapa auricular, según se muestra en la Figura 1.

**Figura 1:** Identificación de puntos auriculares en modelo de silicona, dibujo de la oreja y persona – Coimbra, Portugal, 2025.

Así, la primera parte de la práctica consistió en la localización correcta de los principales puntos auriculares. A continuación, centrándose en la auriculoterapia y sus derivados según la reflexología y la biomedicina, se presentó la inervación de la oreja (nervio aurículo-temporal, vago y auricular mayor) y el mecanismo de acción. Con el fin de aprender la técnica de inspección auricular, la clase se dividió en parejas o tríos, identificando cambios de coloración, morfología y vascularización entre ellos, utilizando focos de luz. A continuación, las parejas practicaron la palpación auricular y la identificación de los puntos.

Después, se presentó un caso clínico para simular la atención, en el que tuvieron que localizar los puntos según la biomedicina y la reflexología y aplicar los puntos utilizando semillas en sus compañeros.

Para profundizar en la auriculoterapia/acupuntura auricular según la MTC, se utilizó como guía un libro^(15)^, que abordaba los diferentes materiales y sus estímulos: armonización (semillas, cristales y pastillas de silicio), tonificación (agujas de 1,0 a 1,5 mm, agujas superficiales y esferas de oro) y sedación (agujas de 1,8 a 2,0 mm, agujas con profundidad y esferas de plata o acero inoxidable). A continuación, se retomó la teoría de los cinco elementos (vista en la etapa teórica) y se trabajaron dos formas de tratamiento según la MTC: (1) Tratamiento del elemento/órgano de choque y (2) Ley de los Ciclos de Generación y Dominancia. Por último, se realizaron simulaciones con casos clínicos y protocolos de tratamiento con el fin de practicar las tres vertientes estudiadas.

### Resultado de la Formación

Después de la formación, en forma de ronda de conversaciones, se evaluó la formación ofrecida y en qué medida las enfermeras y enfermeros se sentían capacitados para aplicar la auriculoterapia/acupuntura auricular. Los *feedback* fueron positivos en cuanto a la calidad de la conducción de las clases y el aprendizaje. Como aspectos negativos, se mencionó la necesidad de una formación con más horas lectivas para el uso de la terapia auricular según la MTC. Además, se debatió la forma de regulación adoptada por el COFEN en Brasil y las vías para una posible regulación de la práctica profesional por parte de la OE en Portugal.

Se mantuvo el grupo de mensajes instantáneos y con frecuencia se producen debates sobre casos, dudas y feedback. Como impacto de la formación, se espera que se produzca una difusión de la auriculoterapia/acupuntura auricular en Portugal, fomentando así nuevas promociones y nuevos horizontes en lo que se refiere a la misma como intervención en la atención de enfermería.

## DISCUSIÓN

El término acupuntura auricular forma parte del ámbito de prácticas de la MTC. No existe consenso sobre el origen de la terapia auricular, que se utiliza desde hace miles de años en diversos países, sin embargo, el mayor desarrollo de la técnica y los primeros documentos registrados proceden de China^(3)^. La auriculoterapia, según el reciente manuscrito de Raphael Nogier, es un término derivado de los estudios de su padre, Paul Nogier, médico francés^(16)^. El término auriculoterapia, en Brasil, se utiliza comúnmente para referirse a cualquier vertiente de la práctica. Cabe destacar que los estudios de Paul Nogier impulsaron el reconocimiento de la auriculoterapia en el contexto internacional^(16)^. Teniendo en cuenta el debate en la comunidad científica sobre la terminología, la opción por utilizar el término auriculoterapia/acupuntura auricular tuvo como objetivo contemplar las diferentes escuelas y vertientes de la práctica, todas ellas con su importancia y relevancia científica.

La MTC es una racionalidad médica compuesta por una “compleja red de interacciones, con algunos momentos de distanciamiento y otros de acercamiento, entre varias escuelas y tradiciones que surgieron a lo largo de la historia”^(17:104)^, y que hoy en día se practica en todo el mundo. Actualmente, la MTC se subdivide en especialidades (similar a la tendencia de la biomedicina), un hecho que puede estar influenciado por la ciencia cartesiana. Sin embargo, hay que recordar que, en su origen, la terapia de la MTC combinaba modalidades de tratamiento como, por ejemplo: acupuntura, masaje, farmacopea, dietoterapia y ejercicios terapéuticos^(17)^.

El documento más importante en el contexto mundial sobre la formación en acupuntura es el *WHO Benchmarks for the training of acupuncture*, elaborado por la OMS en colaboración con más de 50 especialistas de todo el mundo. Este establece categorías de formación en acupuntura, niveles de dificultad de los contenidos y un plan de estudios de los módulos de aprendizaje, que tiene la intersección de contenidos de la MTC frente a las disciplinas básicas del área de la salud^(18)^.

No obstante, lo más destacado de este debate es el reconocimiento del carácter multiprofesional de la acupuntura, de conformidad con la OMS^(19)^, ya que el documento establece una diferenciación en el plan de estudios básico para las profesiones biomédicas, las personas que practican medicinas tradicionales y las personas sin formación biomédica^(18)^. Considerar la acupuntura auricular como una práctica que se contempla en el ámbito de la acupuntura, pero cuya formación puede realizarse de forma independiente, a través de cursos libres (independientemente de la formación en acupuntura), refuerza también su carácter multiprofesional. Las pruebas indican que los enfermeros que trabajan en el área de la MTC han contribuido a mejorar el estado de salud de los pacientes, y que entre las intervenciones utilizadas se encuentra la auriculoterapia/acupuntura auricular^(20)^.

Una investigación que evaluó el perfil de formación de los profesionales de auriculoterapia en Brasil identificó un grupo multidisciplinario de profesionales, en su mayoría mujeres de mediana edad que atendían en sus consultorios privados y con una formación de más de 40 horas, en formato en línea^(21)^. Este aspecto difiere de la investigación brasileña que evaluó solo a enfermeros con formación en auriculoterapia, en la que la mayoría concentra su atención en la APS^(11)^.

En este sentido, considerando el contexto portugués, la Encuesta Social Europea, Ronda 7 (edición 2.0, 2014) identificó que el 14,1 % de la población de Portugal utiliza alguna MCA para el cuidado. Casi una década después, la Encuesta Social Europea, Ronda 11 (edición 2.0, 2023), mostró que los perfiles típicos de los usuarios de MCA en Europa se mantuvieron estables: las mujeres, los grupos de mediana edad y las personas con un alto nivel de escolaridad tienden a utilizarlas más que otros grupos^(22)^.

El ejercicio integrado de las TNC junto con la medicina convencional en el seno del Servicio Nacional de Salud se sugiere en la Ley de Bases de la Salud de 2019, en la base 26, donde se afirma que “El ejercicio de las terapias no convencionales está regulado por la ley, se lleva a cabo de forma integrada con las terapias convencionales y de manera que se garantice la protección de la salud de las personas y las comunidades, la calidad de la asistencia y basándose en la mejor evidencia científica”^(23:63)^.

Una reciente revisión sistemática obtuvo como resultado que la utilidad percibida, definida como los beneficios percibidos de una modalidad de MTCI en la atención de necesidades u objetivos de salud específicos, es el factor más influyente en el uso de la MTCI, según se informa en el 79 % de los estudios. De este modo, los autores sugieren que, al integrar prácticas de MTCI ampliamente aceptadas, las autoridades sanitarias pueden garantizar un uso seguro, estandarizado y basado en la evidencia en entornos médicos. Esta integración puede maximizar el compromiso y la satisfacción del paciente, lo que conduce a mejores resultados de salud^(24)^.

Con ello, analizar experiencias internacionales como las de Brasil^(25)^, China y Estados Unidos de América (EE. UU.) puede fomentar la implementación en otros contextos. En China, la MTC es la práctica principal, y los esfuerzos se centran en traducir las evidencias y desarrollar directrices de práctica clínica, teniendo en cuenta que la MTC ya se ha adoptado en todos los niveles de la asistencia sanitaria. En EE. UU., el sistema de salud está dominado por la medicina convencional, por lo que las intervenciones de implementación se centran en facilitar la prestación de modalidades específicas de MTCI basadas en la evidencia a través de derivaciones de médicos convencionales^(26)^.

En Brasil, teniendo en cuenta las posibilidades y las debilidades de la implementación de la PNPIC, es necesario facilitar la formación y la cualificación de un número adecuado de profesionales para trabajar en el SUS. En este sentido, los autores afirman que el interés de los profesionales en formarse y el número considerable de iniciativas de enseñanza e investigación en las universidades públicas pueden catalizar este proceso^(25)^.

Actualmente, la preocupación de las investigaciones se centra también en la calidad de la formación, según estudios^(11,21,27)^ y los objetivos de la Estrategia Global para 2025–2034^(12)^. Las investigaciones señalan las debilidades en la formación de enfermeros en PICS^(27,28)^, así como la necesidad de directrices mínimas en relación con la formación de profesionales de enfermería en PICS, señalando que la estandarización califica y mejora la seguridad clínica^(7,27)^, aspecto contemplado en el objetivo del informe de experiencia que se basó en una formación estructurada en una directriz inédita elaborada en una tesis.

Estos aspectos corroboran las aspiraciones internacionales, ya que entre los retos mencionados en la Estrategia Global para la MTCI 2025–2034, aprobada en mayo de 2025, en el punto 2.2, se encuentra la necesidad de definir requisitos mínimos de formación^(12)^. Es decir, la preocupación por la calidad de la formación y, en consecuencia, de los planes de estudio y sus requisitos mínimos es un tema cada vez más importante.

Los autores defienden que la recopilación/creación de documentos de referencia para la formación y las prácticas de la *atención sanitaria tradicional, complementaria e integrativa* (*Traditional, complementary and integrative healthcare*- TCIH, por sus siglas en inglés) debe ser un proceso colaborativo entre las organizaciones profesionales implicadas, las instituciones educativas y la OMS/regulador. Los profesionales pueden proporcionar información sobre los resultados educativos necesarios para la profesión, ayudar a recopilar datos sobre seguridad, eficacia y economía, y desempeñar un papel significativo en la regulación de las prácticas^(29)^.

Se necesitan estudios que aborden la realidad de Portugal en el contexto específico de la auriculoterapia/acupuntura auricular. Una investigación reciente tuvo como objetivo identificar las actitudes, los conocimientos y las perspectivas de 4334 médicos portugueses en relación con las TNC, y casi la mitad (45,5%) no se sienten cómodos para hablar, argumentar y explicar sobre las TNC^(30)^. Los autores destacan la necesidad de incluir en los planes de estudios de formación médica más contenidos sobre la eficacia y la seguridad de las TNC, ya que conocerlas es una forma de orientar con seguridad.

Se entiende que estos aspectos pueden trasladarse a la realidad de la enfermería portuguesa, que está directamente implicada en la atención sanitaria. Esto refuerza el carácter pionero del presente informe de experiencia, al desarrollar la primera formación basada en una directriz para la formación de enfermeros en auriculoterapia/acupuntura auricular inédita y científicamente cualificada.

## CONCLUSIÓN

La experiencia pedagógica relatada es inédita e innovadora, ya que se basó en una propuesta de directriz estructurada en el Taller Nacional de Brasil.

Se espera que el presente informe de experiencia fomente el movimiento de regulación de la auriculoterapia/acupuntura auricular como intervención a desarrollar en los servicios como competencia de las enfermeras que hayan realizado la formación mínima, basándose en el modelo brasileño.

## DISPONIBILIDAD DE DATOS

El conjunto de datos que respaldan las conclusiones de este estudio está disponible previa solicitud al autor correspondiente.
